# Cardiac Excrescences of Unusual Origin

**DOI:** 10.1155/2019/8285304

**Published:** 2019-04-11

**Authors:** Jason R. Stuck, Amgad N. Makaryus

**Affiliations:** ^1^PinnacleHealth CardioVascular Institute, 1000 North Front Street, Wormleysburg, PA 17043, USA; ^2^Donald and Barbara Zucker School of Medicine at Hofstra/Northwell, Department of Cardiology, North Shore University Hospital, 300 Community Drive, Manhasset, NY 11030, USA; ^3^Department of Cardiology, NuHealth, Nassau University Medical Center, 2201 Hempstead Turnpike, East Meadow, NY 11554, USA

## Abstract

Mesothelial/monocytic incidental cardiac excrescences (cardiac MICE) are a rare finding that are most often discovered incidentally either upon echocardiography or invasive cardiovascular procedures. In total, less than 50 known cases have been reported since first being discovered over 30 years ago. They are typically benign lesions; however, there has been a reported case of cardiac MICE being responsible for severe cardiopulmonary compromise and another case of the lesion embolizing leading to cerebral infarctions and ultimately death. Cardiac papillary fibroelastomas are also uncommon lesions found in the heart though they are not as rare as cardiac MICE. They are also benign and are typically attached to valvular surfaces; however, they also can be found as mobile masses. Just as cardiac MICE, they are capable of causing turbulent flow and thrombus formation and have been reported as the cause of ischemic events due to their ability to embolize. We present a case of cardiac MICE and cardiac papillary fibroelastoma in an individual who initially presented with neurologic symptoms concerning for a cerebrovascular accident. The patient was found to have a left ventricular mass composed of both cardiac MICE and cardiac papillary fibroelastomas.

## 1. Introduction

Cardiac masses are frequently noted on echocardiogram studies; however, primary neoplastic cardiac tumors are rare. The most frequent etiology of cardiac masses is either a normal variant or a result of an infectious etiology, such as infective endocarditis. The most common cause of a primary cardiac tumor is a myxoma; the second most common is a cardiac lipoma. In spite of being rare, primary cardiac tumors are often thought of, but rarely proven to be, the cause of an embolic event in patients.

Cardiac MICE is a rare primary cardiac mass and is typically a benign incidental finding noticed either through imaging of the heart, invasive cardiac procedures, or at the time of an autopsy. They are solid lesions and are a hypercellular admixture of mesothelial cell clusters, with some degree of mesothelial hyperplasia, histiocytes, fibrin, and macrophages. This mixture is embedded within abundant fibrin, and there are large aggregates of histiocytes and strips of bland mesothelial cells [1, 2].

Their exact origin is not yet confirmed; however, two theories exist to explain their existence. The first hypothesis attests that they are originated by damage to the endocardium, due to trauma or an invasive process such as cardiac catheterization [1–4]. A second hypothesis holds that they are iatrogenically deposited during a cardiac intervention. The masses have most frequently been found in the left cardiac chambers and have been predominantly reported in the left ventricle or on the valves themselves. However, there have been cases describing cardiac MICE in multiple other areas in the heart such as free floating in the left atrium, within an ascending aortic aneurysm, pericardial sac, celiac artery, atrioventricular groove, and right atrium [1, 5].

Cardiac papillary fibroelastomas are rare and are the third most common primary cardiac tumors. They also are found primarily through imaging of the heart, most commonly echocardiography, as well as incidentally during surgery or autopsy. In the majority of the cases of cardiac papillary fibroelastoma, the lesion is found on a valvular surface, most often in the left side of the heart. It has most commonly been reported on the aortic valve (close to half of the time), followed by the mitral valve, then the tricuspid valve, and finally the pulmonary valve. Their origin and pathogenesis are currently not completely understood, but they are hypothesized to be the result of organization of fibrin deposits in the endocardium. As with cardiac MICE, they are free moving masses and therefore have the capability to embolize causing potentially catastrophic events [3, 6].

Here, we discuss the case of an individual who, after presenting with symptoms concerning for a cerebrovascular accident, was found to have a mass in her left ventricle. Pathologic analysis revealed tissue consistent with cardiac MICE and cardiac papillary fibroelastoma.

## 2. Case Summary

The patient is a 77-year-old woman with a past medical history of diabetes, cerebrovascular disease, and coronary artery disease. Her past surgical history is significant for the placement of three coronary stents, which were placed on three separate occasions, the most recent having been placed 6 years prior. The patient initially presented to her primary care physician with “occasional shakes,” which were concerning for a transient ischemic attack. After detecting a carotid bruit on exam, the patient was sent for a bilateral carotid Doppler exam which revealed right carotid artery stenosis. Shortly after this, an echocardiogram was preformed which revealed a 2.0 cm x 0.5 cm mass in the left ventricular outflow tract. The patient was then sent to our institution for further evaluation. After the initial evaluation, the patient was scheduled for carotid endarterectomy.

The surgery was uneventful; however, after the procedure, the patient sustained a cerebrovascular accident. Imaging of the brain suggested an embolic etiology for the cerebrovascular accident, and the patient underwent an echocardiogram to rule out the possibility of a cardiac origin for the embolus. The echocardiogram showed a large mobile mass attached to the undersurface of the posterior mitral apparatus with extension into the left ventricular outflow tract. Additional findings included severe mitral annular calcification, mild to moderate central mitral valve regurgitation with calcified mitral leaflets, calcified trileaflet aortic valve with normal opening, concentric left ventricular remodeling, and an ejection fraction of 70%.

The mass was felt to be at a high risk for remobilization and was considered the cause of her recent cerebrovascular accident. Prior to removing the mass, the patient then underwent coronary angiography which showed severe stenosis of the left anterior descending artery as well as the ostial circumflex artery. Due to her past history of diabetes, as well as multiple vessel disease, the patient and physicians elected to proceed with double vessel coronary artery bypass grafting and concurrent excision of the mass. During the coronary artery bypass, the patient's left anterior descending artery was bypassed using the left internal mammary artery and reversed saphenous vein graft was used to bypass the circumflex marginal branch. An intraoperative transesophageal echocardiogram was completed to better visualize the mass. The findings were an approximately 2.0 cm × 0.5 cm calcified independently mobile echodensity attached to the ventricular side of the mitral annulus extending into the left ventricular outflow tract during ventricular systole. Additional findings included mitral annular calcification and mild-moderate mitral regurgitation, which were nearly identical to the preoperative echocardiogram. The mass was identified by the surgeon at its attachment with the subvalvular chordal apparatus of the posterior mitral leaflet. It was approximately 2.5 cm in diameter and consisted of soft yellowish material with several strands attached to it. Initially, it was thought to be, due to its appearance, either old endocarditis or degenerated calcium.

The mass was resected without complications and sent for pathology. To rule out an infectious etiology of the mass, it was cultured and tested for the presence of acid fast bacilli, all of which were negative. Blood cultures were also sent at this time. The patient was placed on antibiotics due to the possibility of endocarditis, but they were discontinued shortly afterwards as the patient demonstrated no other signs of infection and was afebrile, and all blood cultures were negative. Unexpectedly, the pathology report returned with a diagnosis of cardiac MICE and papillary fibroelastoma. Both of these components comprised the mass that was resected. The patient was discharged and, to date, is alive and doing well.

## 3. Discussion

Our case highlights the unexpected pathologic diagnosis of cardiac MICE. On echocardiography, it is not possible to diagnose cardiac MICE separately from fibroelastoma especially as in this case where the portions/components of the mass were contiguous. In our case, the diagnosis of cardiac MICE was unexpected and was made based on the pathologic analysis of the resected specimen. It is the pathologic findings that allow for the differentiation on the histological level of cardiac MICE from fibroelastoma.

Mesothelial/monocytic incidental cardiac excrescences were first reported in 1979. Due to their apparent origin, the endothelium and its morphologic similarity to histoid hemangiomas found at other locations were initially described as histiocytoid hemangiomas [5, 7–9]. Prior to their discovery, in 1975, Rosai and Dehner reported extravascular lesions, found in 13 different hernia sacs, whose composition was similar to that of MICE and were described as nodular mesothelial hyperplasia. Since that time, additional cases detailing similar lesions have been reported in the pleural and abdominal cavities [10]. In 1997, Chan et al. reported pathologic findings in the pleural cavity and considered the lesions to be nodular mesothelial hyperplasia, induced by inflammatory response as opposed to artifacts, and speculated that a similar hyperplastic change of the mesothelial cells might also occur in the pericardial cavity [7].

The actual term cardiac MICE was not first proposed until a 1994 case report describing four cases resembling a histiocytoid hemangioma was discovered while retrospectively examining surgical pathology files from 1970 to 1992 [1]. Immunohistochemistry concluded that their composition was that of cuboidal cells (exhibiting cytokeratin positivity) and histiocytes (exhibiting CD68 positivity), and examination of the cells for malignancy, by a use of carcinoembryonic antigen and Leu-M1, was negative, leading to the conclusion that the lesions were of benign origin. Additionally, as three out of the four cases had undergone cardiac catheterization, it was first postulated that the lesions were of an iatrogenic nature and in some way related to these previous interventions [1, 11–13].

Since that time, a 2008 article on cardiac MICE reported that the known number of cases were 35, though the true number of cases is uncertain and likely underreported [3]. As more cases of cardiac MICE surfaced, their epidemiology became more complex, but patterns started to emerge. The lesions have typically been found in the left cardiac chambers, with the masses predominantly being detected in the left ventricle or on the valves themselves; however, they have also been reported as free floating in the left atrium, within an ascending aortic aneurysm, pericardial sac, celiac artery, atrioventricular groove, and right atrium [1, 5, 14, 15].

**Figure 1:**
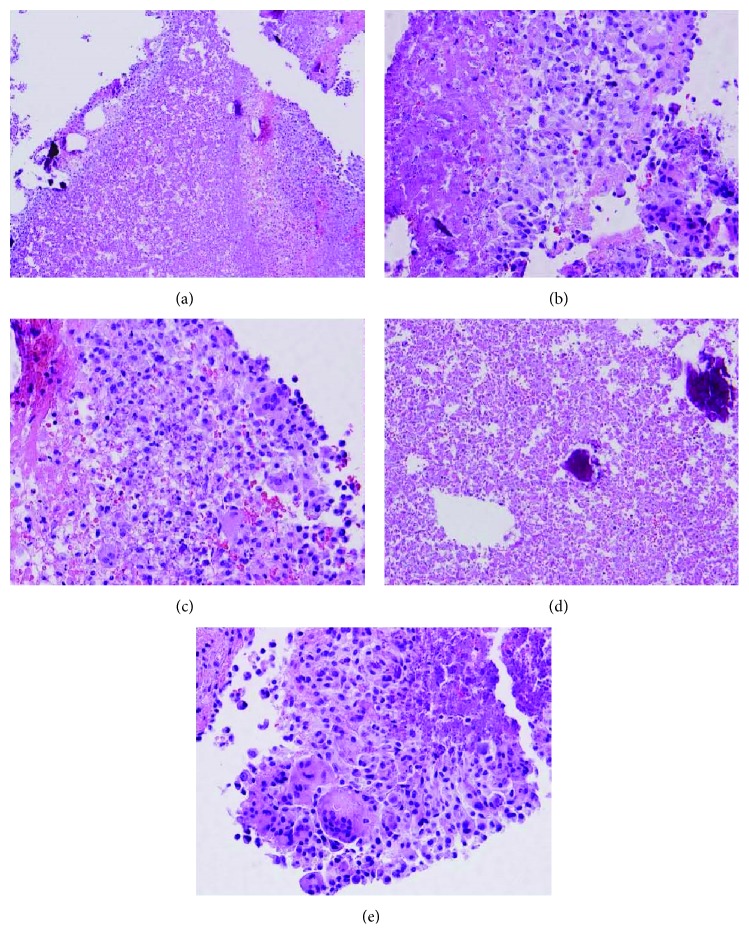
Cardiac MICE.

Pathologically, cardiac MICE (Figure 1) are solid lesions and are a hypercellular admixture of mesothelial cell clusters, with some degree of mesothelial hyperplasia and histiocytes. This mixture is embedded within abundant fibrin, and there are large aggregates of histiocytes and strips of bland mesothelial cells [1, 2].

The origin of cardiac MICE remains up for debate, but two leading theories have arisen to explain their existence, although it is necessary to mention that neither theory by itself is sufficient to fully account for and accurately describe all reported cases of cardiac MICE.

The first theory to be described referred to as the “reactive” theory suggests that these lesions arise secondary to trauma, most often of an iatrogenic etiology. It has been proposed that, after the initial injury to the endocardium occurs, the mesothelial cells are exposed to the bloodstream and aggregate with histiocytes and fibrin beginning the formation of the mass. At least one other case has also been presented that supports the involvement of the adhesion cell process [4, 7, 16]. Of the reported cases of cardiac MICE, a number of them describe cases in which prior instrumentation preceded their discovery and support the reactive theory. It is hypothesized that it was this trauma or microperforation during the instrumentation that lead to mesothelial cell hyperplasia and cell ingrowth from pericardium to endocardium and the development of the lesion [2].

Though prior cardiac intervention is the most common cause of endothelial trauma thus setting into place the chain of events that lead to cardiac MICE formation, at least one case has been reported with a unique underlying catalyst. In 1997, a case of a 38-year-old woman with cardiac MICE was described; although she had no prior cardiac interventions, she was discovered to have a lung adenocarcinoma which involved the hilum but not the pericardium. During surgery, a small fragment of free-floating tissue was found in the pericardial cavity. Upon pathological examination of the specimen, microscopic examination demonstrated a composite of cells indicative of cardiac MICE (clusters of histiocytes, mesothelial cells, and fibrin). In addition to these findings, the sample contained rare pleomorphic adenocarcinoma cells. These cells tested positive for stains confirming their underlying malignant composition. It was then proposed that the surrounding mesothelial cells, histiocytes, and fibrin were formed in response to the invasion of the pericardial space by the adenocarcinoma [2]. In addition, it has been postulated that cardiac MICE may have been induced at least partially by the procoagulant activity of invasive adenocarcinoma. This case again alludes to the concept that adhesion molecules or an adhesive process may play a role in the process [4, 16].

An alternative to the reactive theory is often described as the “artifactual” theory and postulates that cardiac MICE is merely an amalgamation of debris iatrogenically deposited during an invasive cardiac procedure [3, 17]. In 1994, Courtice et al. reported cases of cardiac MICE in which perforation of major cardiac structures at catheterization had not been found in their subject patients [17]. For their study, they examined a material found in extracorporeal bypass pumps and a material adherent to mediastinal and pericardial drains. They found samples consistent with cardiac MICE in 18 of 22 cases of extracorporeal bypass pump filters investigated, and 2 of 15 of mediastinal and pericardial drains following cardiac surgery. They and others suggested that lesions produced during cardiac surgery by the cardiotomy suction and compaction of friable mesothelial strips and other tissue debris and fibrin into tumor-like fragments, may be transported around the operative site on the suction tip. The fragments that are not removed from the cardiac chambers after an intervention are transferred by the suction catheter tips to the intravascular space during heart surgery, and the cellular components may aggregate and form a free-floating mass in the heart [14, 15, 17].

Although most often thought of as an incidental finding, there has been at least one reported case of cardiac MICE causing severe acute cardiopulmonary failure [4]. The patient presented with what initially seemed like a chronic obstructive pulmonary disease exacerbation but decompensated due to severe acute pulmonary edema which was believed to be secondary to severe aortic regurgitation. Prior to the patient's acute decompensation, a transthoracic echocardiogram was completed which showed a 2.0 cm mobile mass on the aortic valve prolapsing into the left ventricular outflow tract. The patient underwent aortic valve repair, and in addition to the mass being visualized on the aortic valve, a soft tissue mass was seen free-floating in the left ventricular outflow tract. Perforation of the left coronary leaflet and adjacent erosion of the endocardium were also noted. Contrary to all previous cases, cardiac MICE in this instance was not incidental, rather it was responsible for the severe acute aortic valve regurgitation, obstruction of the left ventricular outflow tract, and ultimately the patient's severe pulmonary edema and decompensation [4].

Cardiac papillary fibroelastomas are a rare cause of primary cardiac tumors, and studies have estimated that there are approximately 200 cardiac tumors in every 1 million autopsies preformed. Cardiac papillary fibroelastomas are responsible for approximately 7% of all primary cardiac tumors [6, 18–20]. A 2003 report reviewing a total of 725 cases of cardiac papillary fibroelastomas showed that 55% of the patients were male, and although the lesion was most commonly found in the 8^th^ decade of life, there were 10 cases that were reported in children under the age of ten. Of note, the oldest patient was 92 years of age and the youngest case was reported in a newborn child. After analysis of the cases, no clear risk factor was identified for development of cardiac papillary fibroelastomas [6].

**Figure 2:**
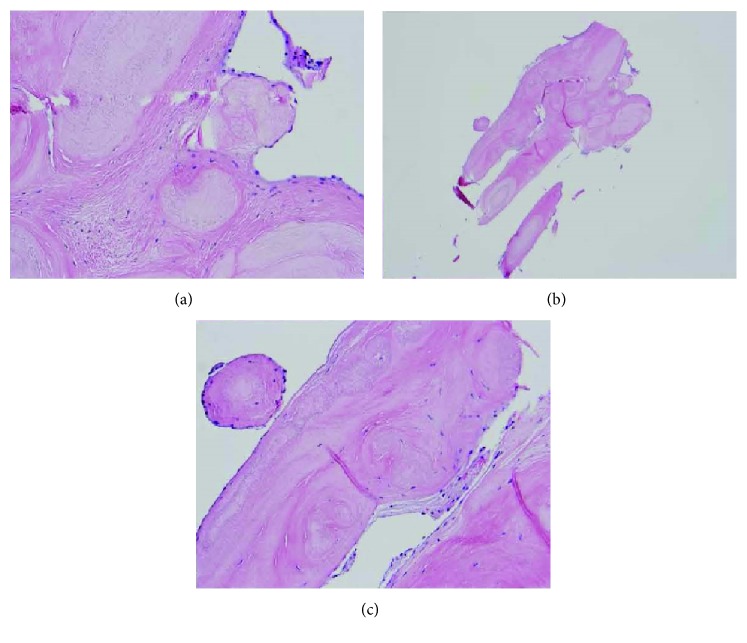
Cardiac papillary fibroelastoma.

Similar to cardiac MICE, cardiac papillary fibroelastomas (Figure 2) are solid lesions most often found on the valves of the heart, and there are multiple theories that exist to suggest their origin. They have been hypothesized to be caused by turbulent blood flow across the endocardium, inflammation, and true neoplasms [21]. Histologically, cardiac papillary fibroelastomas are covered with endothelium that surrounds a layer of acid mucopolysaccharide, and they possess an inner vascular core composed of connective tissue. The connective tissue matrix that comprises the fibroelastoma is a collection of collagen, smooth muscle cells, and elastic fibers [20].

In the vast majority of the cases, nearly 80%, the lesion was found on the valvular surface, most often on the aortic valve (close to half of the time), followed by the mitral valve, then the tricuspid valve, and finally the pulmonary valve. The majority of the cases found that the mass was located in the left heart (>95% of the cases), and therefore, systemic embolization is frequent. Most often incidental findings, the manifestations of cardiac papillary fibroelastomas vary greatly, but the most common presenting manifestation was embolization to systemic, coronary, or cerebral circulation. Other initial presenting symptoms varied and included angina, syncope, and myocardial infarction and sudden death [6].

Cardiac papillary fibroelastomas are most often identified incidentally by echocardiography but other modalities, such as cardiac MRI and cardiac CT, have also been utilized [6]. They have also been incidentally discovered during cardiac catheterization, cardiac surgery, and autopsy [22]. Due to the potential catastrophic events that both cardiac MICE and cardiac papillary fibroelastomas have been associated with, such as sudden death, pulmonary embolism, and myocardial infarction, excision is the recommended treatment. In symptomatic patients, all previous studies have suggested that excision is the preferred treatment and should be offered to all patients who are candidates. Surgery is curative and has been shown to improve both the long and short term prognosis, and recurrence has yet to be reported in regard to both lesions [6]. If the patient is not a candidate for surgery, oral anticoagulation is the recommended treatment for cardiac papillary fibroelastomas, but there have been no randomized controlled trials to date on the efficacy of this treatment [6, 23, 24]. No previous studies have been conducted, and no interventions currently exist for cardiac MICE in patients that are not surgical candidates. Additionally, as mentioned, the challenge remains the difficulty in the echocardiographic diagnosis of cardiac MICE which leads to the necessity of cardiac MICE diagnosis based on the histopathologic specimen analysis of the resected mass.

This case illustrates a rare case of a lesion comprised of both cardiac MICE and cardiac papillary fibroelastoma. To our knowledge, although they have been presented separately, there has never been a case reported of a mass consisting of both cardiac MICE and cardiac papillary fibroelastoma. Additionally, there have been very few cases of cardiac MICE embolizing causing systemic symptoms. In the case of our patient, the likely origin of her neurologic symptoms was an embolic event caused by her left ventricular mass. The patient did have a history of prior cardiac instrumentation as she underwent cardiac catheterization on three separate occasions which would support the reactive theory of cardiac MICE and potentially an inflammatory/reactive etiology of cardiac papillary fibroelastomas.

## References

[1] Veniot J. P., Tazelaar H. D., Edwarda W. D., Colby T. V. (1994). Mesothelial/monocytic incidental cardiac excrescences: cardiac MICE. *Modern Pathology*.

[2] Argani P., Sternberg S. S., Burt M., Adsay N. V., Klimstra D. S. (1997). Metastatic adenocarcinoma involving a mesothelial/monocytic incidental cardiac excrescence (cardiac MICE). *The American Journal of Surgical Pathology*.

[3] Censi S., Dell'Amore A., Conti R., Lorenzini P. (2008). Cardiac mesothelial/monocytic-incidental-excrescence: more than an artifactual lesion?. *Interactive CardioVascular and Thoracic Surgery*.

[4] Pham T. T., Antons K., Shishido R., Mullvain J., Salem F., Haghighi P. (2005). A case of mesothelial/monocytic cardiac excrescence causing severe acute cardiopulmonary failure. *The American Journal of Surgical Pathology*.

[5] Rosai J., Gold J., Landy R. (1979). The histiocytoid hemangiomas: a unifying concept embracing several previously described entities of skin, soft tissue, large vessels, bone and heart. *Human Pathology*.

[6] Gowda R. M., Khan I. A., Nair C. K., Mehta N. J., Vasavada B. C., Sacchi T. J. (2003). Cardiac papillary fibroelastoma: a comprehensive analysis of 725 cases. *American Heart Journal*.

[7] Chan J. K. C., Loo K. T., Yau B. K. C., Lam S. Y. (1997). Nodular histiocytic/mesothelial hyperplasia: a lesion potentially mistaken for a neoplasm in transbronchial biopsy. *The American Journal of Surgical Pathology*.

[8] Dannaker C., Piacquadio D., Willoughby C. B., Goltz R. W. (1989). Histiocytoid hemangioma: a disease spectrum. Report of a case with simultaneous cutaneous and bone involvement limited to one extremity. *Journal of the American Academy of Dermatology*.

[9] Luthringer D. J., Virmani R., Weiss S. W., Rosai J. (1990). A distinctive cardiovascular lesion resembling histiocytoid (epithelioid) hemangioma evidence suggesting mesothelial participation. *The American Journal of Surgical Pathology*.

[10] Rosai J., Dehner L. P. (1975). Nodular mesothelial hyperplasia in hernia sacs: a benign reactive condition simulating a neoplastic process. *Cancer*.

[11] Wu M., Anderson A., Kahn L. B. (2000). A report of mesothelial/monocytic incidental cardiac excrescences and a literature review. *Annals of diagnostic pathology*.

[12] Rubin M. A., Snell J. A., Tazelaar H. D., Lack E. E., Austenfeld J. L., Azumi N. (1995). Cardiac papillary fibroelastoma: an immunohistochemical investigation and unusual clinical manifestations. *Modern Pathology*.

[13] Ryu S. W., Beom M. S., Kim S. R., Kim G. S. (2015). Cardiac papillary fibroelastoma. *Revista Portuguesa de Cardiologia*.

[14] Ikeda Y., Yutani C., Imakita M. (1998). Two cases of mesothelial/monocytic incidental cardiac excrescences of the heart. *Pathology International*.

[15] Lin C. Y., Tsai F. C., Fang B. R. (2005). Mesothelial/monocytic cardiac incidental excrescences of the heart: case report and literature review. *International Journal of Clinical Practice*.

[16] Suarez-Vilela D., Izquierdo-Garcia F. M. (2005). Mesothelial/Monocytic Incidental Cardiac Excrescence: A Process Mediated by Adhesion Molecules?. *The American Journal of Surgical Pathology*.

[17] Courtice R. W. (1994). Tissue fragments recovered at cardiac surgery masquerading as tumoral proliferations: evidence suggesting iatrogenic or artefactual origin and common occurrence. *The American Journal of Surgical Pathology*.

[18] Reynen K. (1996). Frequency of primary tumors of the heart. *The American Journal of Cardiology*.

[19] Goldberg H. P., Glenn F., Dotter C. T., Steinberg I. (1952). Myxoma of the left atrium: diagnosis made during life with operative and postmortem findings. *Circulation*.

[20] Sun J. P., Asher C. R., Yang X. S. (2001). Clinical and echocardiographic characteristics of papillary fibroelastomas: a retrospective and prosepective study in 162 patients. *Circulation*.

[21] Costa M. J., Makaryus A. N., Rosman D. R. (2006). A rare case of a cardiac papillary fibroelastoma of the pulmonary valve diagnosed by echocardiography. *The International Journal of Cardiovascular Imaging*.

[22] Edwards F. H., Hale D., Cohen A., Thompson L., Pezzella A. T., Virmani R. (1991). Primary cardiac valve tumors. *The Annals of Thoracic Surgery*.

[23] Pinelli G., Carteaux J. P., Mertes P. M., Civit T., Trinh A., Villemot J. P. (1995). Mitral valve tumor revealed by stroke. *The Journal of Heart Valve Disease*.

[24] Sastre-Garriga J., Molina C., Montaner J. (2000). Mitral papillary fibroelastoma as a cause of cardiogenic embolic stroke: report of two cases and review of the literature. *European Journal of Neurology*.

